# Urinary Excretion of *N*^1^-methyl-2-pyridone-5-carboxamide and *N*^1^-methylnicotinamide in Renal Transplant Recipients and Donors

**DOI:** 10.3390/jcm9020437

**Published:** 2020-02-06

**Authors:** Carolien P. J. Deen, Anna van der Veen, António W. Gomes-Neto, Johanna M. Geleijnse, Karin J. Borgonjen-van den Berg, M. Rebecca Heiner-Fokkema, Ido P. Kema, Stephan J. L. Bakker

**Affiliations:** 1 Department of Internal Medicine, University of Groningen, University Medical Center Groningen, 9713 GZ Groningen, The Netherlands; a.w.gomes.neto@umcg.nl (A.W.G.-N.); s.j.l.bakker@umcg.nl (S.J.L.B.); 2 Department of Laboratory Medicine, University of Groningen, University Medical Center Groningen, 9713 GZ Groningen, The Netherlands; a.van.der.veen03@umcg.nl (A.v.d.V.); m.r.heiner@umcg.nl (M.R.H.-F.); i.p.kema@umcg.nl (I.P.K.); 3 TiFN, 6709 PA Wageningen, The Netherlands; 4 TransplantLines Food and Nutrition Biobank and Cohort Study, University of Groningen, University Medical Center Groningen, 9713 GZ Groningen, The Netherlands; 5 Division of Human Nutrition and Health, Wageningen University, 6708 PB Wageningen, The Netherlands; marianne.geleijnse@wur.nl (J.M.G.); karin.borgonjen@wur.nl (K.J.B.-v.d.B.)

**Keywords:** *N*^1^-methyl-2-pyridone-5-carboxamide, *N*^1^-methylnicotinamide, urinary excretion, renal transplantation, kidney function, biomarker, niacin status, tryptophan, vitamin B_3_

## Abstract

*N*^1^-methylnicotinamide (*N*^1^-MN) and *N*^1^-methyl-2-pyridone-5-carboxamide (2Py) are successive end products of NAD^+^ catabolism. *N*^1^-MN excretion in 24-h urine is the established biomarker of niacin nutritional status, and recently shown to be reduced in renal transplant recipients (RTR). However, it is unclear whether 2Py excretion is increased in this population, and, if so, whether a shift in excretion of *N*^1^-MN to 2Py can be attributed to kidney function. Hence, we assessed the 24-h urinary excretion of 2Py and *N*^1^-MN in RTR and kidney donors before and after kidney donation, and investigated associations of the urinary ratio of 2Py to *N*^1^-MN (2Py/*N*^1^-MN) with kidney function, and independent determinants of urinary 2Py/*N*^1^-MN in RTR. The urinary excretion of 2Py and *N*^1^-MN was measured in a cross-sectional cohort of 660 RTR and 275 healthy kidney donors with liquid chromatography-tandem mass spectrometry (LC-MS/MS). Linear regression analyses were used to investigate associations and determinants of urinary 2Py/*N*^1^-MN. Median 2Py excretion was 178.1 (130.3–242.8) μmol/day in RTR, compared to 155.6 (119.6–217.6) μmol/day in kidney donors (*p* < 0.001). In kidney donors, urinary 2Py/*N*^1^-MN increased significantly after kidney donation (4.0 ± 1.4 to 5.2 ± 1.5, respectively; *p* < 0.001). Smoking, alcohol consumption, diabetes, high-density lipoprotein (HDL), high-sensitivity C-reactive protein (hs-CRP) and estimated glomerular filtration rate (eGFR) were identified as independent determinants of urinary 2Py/*N*^1^-MN in RTR. In conclusion, the 24-h urinary excretion of 2Py is higher in RTR than in kidney donors, and urinary 2Py/*N*^1^-MN increases after kidney donation. As our data furthermore reveal strong associations of urinary 2Py/*N*^1^-MN with kidney function, interpretation of both *N*^1^-MN and 2Py excretion may be recommended for assessment of niacin nutritional status in conditions of impaired kidney function.

## 1. Introduction

Niacin, or vitamin B_3_, is the precursor of the nicotinamide nucleotide coenzyme NAD^+^. An adequate niacin status is vital to provide reducing equivalents for energy metabolism, and substrates of NAD^+^ consuming enzymes, including adenosine diphosphate (ADP)-ribosyl transferases and deacetylases, that transfer ADP-ribose moieties from NAD^+^ and NADP^+^ [1,2].

**Figure 1:**
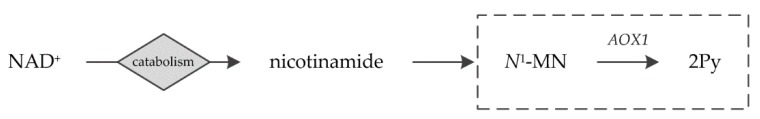
Schematic overview of NAD^+^ catabolism. 2Py is the end product of NAD^+^ catabolism after AOX1-dependent oxidation of *N*^1^-MN, framed by the dotted line. AOX1, aldehyde oxidase; *N*^1^-MN, *N*^1^-methylnicotinamide; 2Py, *N*^1^-methyl-2-pyridone-5-carboxamide.

Niacin nutritional status is most commonly assessed by the 24-h urinary excretion of *N*^1^-methylnicotinamide (*N*^1^-MN) as a breakdown product of NAD^+^, and recommended as such by authorities, including the WHO and the European Food Safety Authority (EFSA) [3,4]. However, *N*^1^-methyl-2-pyridone-5-carboxamide (2Py) is the end product of NAD^+^ catabolism, after aldehyde oxidase (AOX1)-dependent oxidation of *N*^1^-MN (Figure 1) [5,6]. Although the 24-h urinary excretion of *N*^1^-MN has shown the most sensitive response to oral test doses of niacin equivalents [3,7], excretion of 2Py, whether or not combined with that of *N*^1^-MN, has also been implicated for the assessment of niacin status [8,9,10,11].

In a recent study, we found that *N*^1^-MN excretion is lower in renal transplant recipients (RTR) than in healthy controls [12]. As this discrepancy could not be explained by lower dietary intake of niacin equivalents, enhanced enzymatic conversion of *N*^1^-MN to 2Py by AOX1 might be present in this population due to the suggested contribution of AOX1 to *N*^1^-MN clearance with lower kidney function [13,14]. It is unclear whether 2Py excretion is increased in RTR, and if so, whether a shift in excretion of *N*^1^-MN to 2Py can be attributed to kidney function.

Hence, to evaluate the applicability of *N*^1^-MN excretion as a biomarker of niacin nutritional status in conditions of impaired kidney function, we measured the 24-h urinary excretion of 2Py and *N*^1^-MN in RTR and kidney donors before and after kidney donation, allowing us to (1) compare the 24-h urinary excretion of 2Py in RTR and kidney donors, (2) investigate the effect of kidney donation on the excretion of 2Py and *N*^1^-MN in kidney donors, (3) assess whether the urinary ratio of 2Py to *N*^1^-MN (2Py/*N*^1^-MN) is associated with kidney function, and (4) identify determinants of urinary 2Py/*N*^1^-MN in RTR.

## 2. Materials and Methods

### 2.1. Study Population

This cross-sectional study was based on a well-characterized, single-center cohort of 707 RTR (aged ≥18 years) who visited the outpatient clinic of the University Medical Center Groningen, Groningen, the Netherlands, between 2008 and 2011, with a functioning graft for at least 1 year and no history of alcohol and/or drug abuse [15,16,17]. As a control group, 367 healthy kidney donors were included who participated in a screening program before kidney donation, and of whom biomaterial was collected before and, after declared eligible, 3 months after kidney donation. Exclusion of subjects with missing biomaterial or niacin supplementation use left 660 RTR and 275 kidney donors, of which 85 underwent donor nephrectomy during the inclusion period, eligible for statistical analyses. Signed informed consent was obtained from all participating subjects and the study protocol was approved by the institutional review board (METc 2008/186) adhering to the Declaration of Helsinki. This study included the same cohort of 660 RTR and 275 kidney donors for data collection as reported previously [12].

### 2.2. Data Collection and Measurements

Participants were instructed to collect a 24-h urine sample on the day before their morning visit to the outpatient clinic, and to fast overnight for 8 to 12 h. Urine samples were collected under oil, and chlorhexidine was added as an antiseptic agent. Fasting blood samples were drawn after completion of the urine collection. Laboratory measurements were performed directly with spectrophotometric-based routine clinical laboratory methods (Roche Diagnostics, Rotkreuz, Switzerland). Body composition and hemodynamic parameters were measured according to a previously described, strict protocol [15]. Diabetes was diagnosed if fasting plasma glucose was ≥7.0 mmol/L or antidiabetic medication was used. Proteinuria was diagnosed if total urinary protein excretion was ≥0.5 g/day as measured by a biuret reaction-based assay (MEGA AU510; Merck Diagnostica, Darmstadt, Germany).

Dietary intake was assessed with a validated semi-quantitative food frequency questionnaire (FFQ) [18,19]. The self-administered questionnaire was filled out at home and inquired about 177 food items over the last month. During the outpatient clinic visit, the FFQ was checked for completeness by a trained researcher and inconsistent answers were verified with the participant. The FFQ was validated for RTR as previously reported [16]. Dietary data were converted into daily nutrient intake using the Dutch Food Composition Table of 2006 [20]. Intake of niacin equivalents was calculated by adding up niacin and one-sixtieth of tryptophan intake. Subjects who were using niacin supplementation were excluded. Smoking behavior was assessed with a separate questionnaire [21]. Data on medication and vitamin supplements use, and medical history were obtained from medical records [21].

The estimated glomerular filtration rate (eGFR) was calculated by the combined creatinine and cystatin C-based Chronic Kidney Disease Epidemiology Collaboration equation [22], which has shown to be the most accurate equation in RTR [23]. The glomerular filtration rate (GFR) was measured by infusion of ^125^I-Iothalamate as described previously [24].

### 2.3. Assessment of 2Py and N^1^-MN Excretion

Measurement of 2Py and *N*^1^-MN concentrations was performed with a validated liquid chromatography (Luna HILIC column; Phenomenex, Torrance, CA, USA) isotope dilution-tandem mass spectrometry (Quattro Premier; Waters, Milford, MA, USA) (LC-MS/MS) method, as described previously [25], with the addition of *N*^1^-methyl-2-pyridone-5-carboxamide-d_3_ in acetonitrile as an internal standard. The 24-h urinary excretion of 2Py and *N*^1^-MN (μmol/day) was obtained by multiplying concentrations (μmol/L) by total urine volume calculated from weight (L/day).

### 2.4. Statistical Analysis

Data are presented as the mean ± SD, median (IQR) and absolute number (percentage) for normally distributed, skewed and nominal data, respectively. Assumptions for normality were checked by visual judgments of the corresponding frequency distribution and Q-Q plot.

Baseline characteristics of RTR and the total cohort of kidney donors were compared by means of *t*, Mann–Whitney, and Chi-Square tests, of which age, sex, body surface area, *N*^1^-MN excretion and eGFR have been reported previously [12]. Crude associations of 2Py and *N*^1^-MN excretion with intake of niacin equivalents were investigated with linear regression analyses. Characteristics of kidney donors before and after kidney donation were compared by means of paired samples *t* and Wilcoxon signed rank tests.

Linear regression analyses were used to investigate associations of urinary 2Py/*N*^1^-MN with kidney function in RTR and kidney donors, with additional adjustments for age and sex. Effect modification between either age or sex and kidney function with urinary 2Py/*N*^1^-MN was assessed by including the corresponding cross product term in the linear regression model.

Linear regression analyses were furthermore employed to investigate cross-sectional associations of urinary 2Py/*N*^1^-MN with baseline variables in RTR. Variables were 2-base log-transformed when assumptions of normality and homogeneity of variance of the residuals, based on visual judgement of P-P and scatter plots, respectively, were not met. Multivariable linear regression analyses were used to identify determinants of urinary 2Py/*N*^1^-MN, by entering terms with *p*-value <0.1 in univariable analysis, and eliminating the least significant term stepwise until the remaining terms contributed significantly to the model.

For all statistical analyses, a two-sided *p*-value of less than 0.05 was considered to indicate statistical significance and SPSS Statistics version 23.0 (IBM, Armonk, NY, USA) was used as software.

## 3. Results

### 3.1. Excretion of 2Py in Kidney Donors and RTR

The total cohort consisted of 660 stable RTR (57% male; mean age 53.0 ± 12.7 years), included at a median time of 5.6 (2.0–12.0) years after transplantation, and 275 healthy kidney donors (41% male; mean age 53.3 ± 10.7 years) (Table 1). Differences in the 24-h urinary excretion of 2Py and *N*^1^-MN and kidney function are shown in Table 1. 2Py excretion was higher in RTR than in kidney donors (178.1 (130.3–242.8) versus 155.6 (119.6–217.6) μmol/day, respectively; *p* < 0.001), while *N*^1^-MN excretion was lower in RTR than in kidney donors (22.0 (15.8–31.8) versus 41.4 (31.6–57.2) μmol/day, respectively; *p* < 0.001). Kidney function was significantly lower in RTR than in kidney donors (eGFR: 45.8 ± 18.7 versus 91.0 ± 14.2 mL/min/1.73 m^2^, respectively; *p* < 0.001 and GFR: 52.4 ± 17.4 versus 82.3 ± 29.7 mL/min/1.73 m^2^, respectively; *p* < 0.001). Urinary 2Py/*N*^1^-MN was significantly higher in RTR than in kidney donors (8.7 ± 3.8 versus 4.0 ± 1.4, respectively; *p* < 0.001), while the sum of 2Py and *N*^1^-MN excretion was similar (198.3 (155.9–269.4) versus 203.7 (149.4–274.7) μmol/day, respectively; *p* = 0.98). The urinary fraction of 2Py was higher in RTR than in kidney donors (89.1% (86.4%–91.3%) versus 79.0% (75.6%–82.1%), respectively; *p* < 0.001), and that of *N*^1^-MN was lower (10.9% (8.7%–13.6%) versus 21.0% (17.9%–24.4%), respectively; *p* < 0.001). The 24-h urinary excretion of 2Py, *N*^1^-MN and the sum of 2Py and *N*^1^-MN, but not urinary 2Py/*N*^1^-MN, were directly associated with intake of niacin equivalents (Table S1).

### 3.2. Excretion of 2Py and N^1^-MN before and after Kidney Donation in Kidney Donors

**Figure 2:**
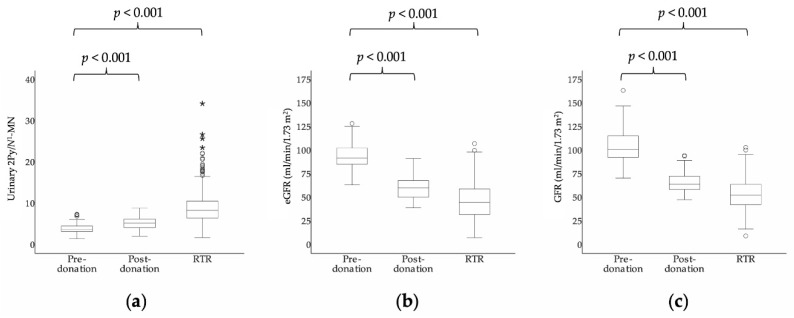
Box plots of (**a**) urinary 2Py/*N*^1^-MN, (**b**) eGFR and (**c**) GFR in kidney donors before (*n* = 85) and after kidney donation (*n* = 85) and RTR (*n* = 660), respectively. Boxes, bars and whiskers represent IQRs, medians and values <1.5 × IQR, respectively, whereas outliers (1.5–3 × IQR) are indicated by circles and extreme outliers (>3 × IQR) by asterisks. *p*-value for difference between kidney donors before and after kidney donation was tested by paired samples *t* and Wilcoxon signed rank tests for normally and skewed distributed continuous variables, respectively. *p*-value for difference between RTR and kidney donors before donation was tested by *t* and Mann–Whitney tests for normally and skewed distributed continuous variables, respectively. eGFR, estimated glomerular filtration rate; GFR, glomerular filtration rate; *N*^1^-MN, *N*^1^-methylnicotinamide; RTR, renal transplant recipients; 2Py, *N*^1^-methyl-2-pyridone-5-carboxamide; 2Py/*N*^1^-MN, ratio of 2Py to *N*^1^-MN.

The 24-h urinary excretion of 2Py and *N*^1^-MN and kidney function in 85 kidney donors before and after kidney donation are shown in Table 1 and Figure 2. At a median time of 1.64 (1.61–1.87) months after kidney donation, 2Py excretion did not change significantly (152.8 (124.4–215.1) to 161.7 (116.6–227.8) μmol/day, respectively; *p* = 0.31), while *N*^1^-MN decreased (40.9 (31.0–58.2) to 32.5 (23.4–44.0) μmol/day, respectively; *p* < 0.001). Kidney function decreased significantly after kidney donation (eGFR: 92.8 ± 13.9 to 60.1 ± 12.1 mL/min/1.73 m^2^, respectively; *p* < 0.001 and GFR: 103.7 ± 16.7 to 65.3 ± 10.4 mL/min/1.73 m^2^, respectively; *p* < 0.001). Urinary 2Py/*N*^1^-MN increased after kidney donation (4.0 ± 1.4 to 5.2 ± 1.5, respectively; *p* < 0.001), while the sum of 2Py and *N*^1^-MN excretion did not change (198.3 (162.3–270.8) to 189.7 (141.9–271.6) μmol/day, respectively; *p* = 0.90). The urinary fraction of 2Py increased after kidney donation (78.3% (75.5%–81.8%) to 83.5% (80.0%–86.0%), respectively; *p* < 0.001), and that of *N*^1^-MN decreased (21.7% (18.2%–24.5%) to 16.5% (14.0%–20.0%), respectively; *p* < 0.001).

### 3.3. Associations of Urinary 2Py/N^1^-MN with Kidney Function

Urinary 2Py/*N*^1^-MN was associated with kidney function in RTR (eGFR: *β* = −0.40; *p* < 0.001 and GFR: *β* = −0.39; *p* < 0.001) and the total cohort of kidney donors (eGFR: *β* = −0.17; *p* = 0.03 and GFR: *β* = −0.20; *p* = 0.003), but not in the pre- (eGFR: *β* = −0.01; *p* = 0.94 and GFR: *β* = −0.02; *p* = 0.89) and post-donation subgroups of kidney donors (eGFR: *β* = −0.11; *p* = 0.42 and GFR: *β* = 0.15; *p* = 0.27), with adjustment for age and sex (Table 2). No significant interaction between either age or sex with kidney function was found in the association with urinary 2Py/*N*^1^-MN in RTR and kidney donors.

### 3.4. Characteristics and Associations with Urinary 2Py/N^1^-MN in RTR

Characteristics of the RTR cohort are shown in Table 3. Urinary 2Py/*N*^1^-MN was positively associated with body surface area, body mass index (BMI), glucose homeostasis parameters, triglycerides, mean arterial pressure, high-sensitivity C-reactive protein (hs-CRP), proteinuria, and use of antidiabetics, antihypertensives, acetylsalicylic acid, proton pump inhibitors and tacrolimus. Inverse associations were found between urinary 2Py/*N*^1^-MN and smoking, alcohol consumption, energy intake, vitamin B_6_ intake, high-density lipoprotein (HDL) and eGFR.

### 3.5. Determinants of Urinary 2Py/N^1^-MN in RTR

Stepwise multivariable linear regression analyses with backward elimination revealed smoking, alcohol consumption, diabetes, HDL, hs-CRP and eGFR as independent determinants of urinary 2Py/*N*^1^-MN in RTR (Table 4). In the final model, urinary 2Py/*N*^1^-MN was positively associated with diabetes (β = 0.10; *p* = 0.01) and hs-CRP (β = 0.10; *p* = 0.009), and inversely associated with smoking (β = −0.13; *p* = 0.001), alcohol consumption (β = −0.12; *p* = 0.002), HDL (β = −0.12; *p* = 0.002) and eGFR (β = −0.38; *p* < 0.001).

## 4. Discussion

This study aimed to investigate the 24-h urinary excretion of both 2Py and *N*^1^-MN as major catabolic products of NAD^+^ with regard to kidney function. We assessed 2Py and *N*^1^-MN excretion in RTR and healthy kidney donors as a model of renal disease, and in kidney donors before and after unilateral nephrectomy as a model of isolated renal function impairment. In RTR, 2Py excretion was significantly higher compared to that in kidney donors. Urinary 2Py/*N*^1^-MN increased significantly in kidney donors after donation. In both RTR and kidney donors, urinary 2Py/*N*^1^-MN was associated with kidney function. Kidney function was furthermore revealed as the strongest determinant of urinary 2Py/*N*^1^-MN in RTR.

NAD^+^ is formed either de novo from tryptophan via the kynurenine pathway, or via salvage pathways from preformed nicotinamide, nicotinic acid and nicotinamide riboside [26], commonly known as niacin, or vitamin B_3_. NAD^+^ catabolism proceeds via nicotinamide and its downstream metabolites *N*^1^-MN and 2Py, respectively (Figure 1), and these products are found in both plasma and urine [27]. *N*^1^-MN itself exhibits anti-inflammatory properties, and is produced by muscle in response to hypoxia and depletion of energy stores, besides its primary production in the liver [28]. Whereas nicotinamide is reabsorbed by renal tubules and only small amounts appear in urine, *N*^1^-MN and 2Py account for 20%–35% and 45%–60%, respectively, of all urinary NAD^+^ metabolites [29]. The WHO and the EFSA recommend the 24-h urinary excretion of *N*^1^-MN for laboratory assessment of niacin nutritional status accordingly [3,4]. In a previous study, we found that *N*^1^-MN excretion is clearly reduced in RTR compared to healthy kidney donors [12]. The fact that this is paralleled by a significant elevation of 2Py excretion in the present study, raises speculation that enhanced enzymatic conversion of *N*^1^-MN to 2Py by AOX1 may be present in RTR. Furthermore, the opposing shifts of 2Py and *N*^1^-MN excretion in kidney donors after donation, may imply a putative isolated effect of renal function impairment on urinary 2Py/*N*^1^-MN.

Regarding kidney function, urinary 2Py/*N*^1^-MN was positively associated with kidney function in both RTR and the total cohort of kidney donors. Renal clearance of *N*^1^-MN is affected by lower kidney function [13,14], being freely filtered at the glomerulus and tubular secreted, with negligible and saturable tubular reabsorption [30,31]. 2Py has previously been classified as a uremic retention product by the European Uremic Toxin Working Group [32,33], though specific mechanisms of its renal clearance have yet not been characterized. Whereas plasma concentrations of 2Py are reported to increase progressively with chronic kidney disease stages [34], those of *N*^1^-MN are suggested to be less sensitive to kidney function because of the contribution of AOX1 to *N*^1^-MN clearance [13,14]. In view of this, we can speculate upon slower excretion of *N*^1^-MN, hence prolonged exposure to 2Py-forming AOX1 that is related to kidney function, rather than retention of 2Py primarily. This speculation is supported by the fact that kidney function appeared to have only a minor effect on the daily excretion of the sum of 2Py and *N*^1^-MN in all groups. The presence of a significant association of urinary 2Py/*N*^1^-MN with kidney function in the total cohort of kidney donors, but not in the pre- and post-donation subgroups, is most likely due to smaller effect sizes in the latter subgroups of kidney donors being declared eligible after pre-donation screening.

The identification of eGFR as the strongest independent determinant of urinary 2Py/*N*^1^-MN in RTR further supports the notion of an isolated effect of kidney function. Other identified determinant factors include those that are known to affect the enzymatic activity of the aforementioned 2Py-forming AOX1 and most likely contribute as such. In fact, urinary 2Py/*N*^1^-MN has been used as an index to estimate in vivo AOX1 levels and activity [35], being regulated by a wide variety of endogenous and exogenous factors [36]. Smoking and alcohol consumption are well-known factors [37,38] that showed an inverse association with urinary 2Py/*N*^1^-MN in RTR. Diabetes and inflammatory mediators [37], including hs-CRP [39,40], have also been implicated in AOX1 activity, as well as HDL-cholesterol-levels via interaction of AOX1 with the ATP-binding cassette transporter A1 (ABCA1) which is a regulator of HDL metabolism [41,42]. Surprisingly, medication use did not appear to affect urinary 2Py/*N*^1^-MN in RTR, despite the significant function of AOX1 in metabolizing xenobiotics. Importantly, the fact that urinary 2Py/*N*^1^-MN has multiple determinants in addition to eGFR, precludes its use as a biomarker of kidney function.

Excessive poly (ADP-ribose) polymerase (PARP) activation induced by stressors such as inflammation, oxidative stress and DNA damage that are predominant in RTR [43,44], has also been implicated in higher production of 2Py from NAD^+^ degradation [45,46]. One would, however, expect that this would be reflected by an overall increase of NAD^+^ catabolites, which is opposed by the two-fold reduction of *N*^1^-MN excretion in our RTR population.

In general, higher urinary output of NAD^+^ metabolites indicates higher niacin nutritional status, being excreted after the pool of pyridine nucleotide coenzymes is filled [47]. Acute stress may alter this output, but not steady state conditions, in which elimination and production rates are equal [48]. However, the ratio of metabolites is subject to factors that affect not only the activity of 2Py-forming AOX1, but according to our data also kidney function. In a previous study, we found *N*^1^-MN excretion to be lower in RTR independent of dietary intake of niacin equivalents, as well as to be positively associated with kidney function [12]. According to the present study, the latter association remains when taking into account 2Py excretion, by means of urinary 2Py/*N*^1^-MN. Therefore, although urinary excretion of *N*^1^-MN is the most common and recommended index [3,4], our findings suggest that this index might be of limited value in conditions of kidney function impairment and future studies may confirm whether 2Py excretion should at least be additionally interpreted for evaluation of niacin nutritional status.

The speculative presence of slower excretion, hence prolonged exposure of *N*^1^-MN to 2Py-forming AOX1 with kidney function impairment has not been confirmed in previous studies. In fact, this speculation indicates straight substrate conversion kinetics, which is unlikely to fully account for the previously reported, increased serum concentrations of 2Py in patients with chronic renal failure [46]. More specifically, Rutkowski et al. suggested high serum concentrations of 2Py in chronic renal failure to be a result of kidney function impairment, based on the fact that serum concentrations of 2Py were approximately 20-fold higher in patients with advanced renal failure than in healthy subjects (15.5 ± 5.8 µmol/L versus 0.83 ± 0.18 µmol/L), with only a transient drop after dialysis, and a permanent reduction after kidney transplantation [46]. Accordingly, given its accumulation, along with a deterioration of kidney function, and its toxic properties due to significant inhibition of PARP activity, 2Py has been identified as a uremic toxin [32,45,46]. As we only measured urinary excretion of 2Py, it cannot be ruled out whether increased urinary excretion of 2Py is solely the consequence of increased serum concentrations of 2Py, due to decreased renal clearance, rather than conversion kinetics of *N*^1^-MN to 2Py by AOX1, and future studies are warranted to address this matter.

Strengths of this study include the large sample size of a specific patient group and the availability of healthy kidney donors before donation as a control group, and after donation as a model of isolated renal function impairment. Moreover, the extensive characterization of RTR allowed us to control for other factors that could affect 2Py/*N*^1^-MN in 24-h urine, and to comprehensively identify determinants of urinary 2Py/*N*^1^-MN. The ratio of metabolites in 24-h urine provides a measure to demonstrate changes in metabolism related to renal function, while being the least sensitive to 24-h urine collection errors. Limitations of this study are its observational nature, which prohibits causal inferences, as well as final conclusions on underlying mechanisms of increased urinary 2Py/*N*^1^-MN in RTR and kidney donors after kidney donation, and associations with kidney function. Therefore, it remains to be determined whether the association of urinary 2Py/*N*^1^-MN with kidney function is a causal relation. The observational design of this study did neither allow us to rule out increased serum concentrations of 2Py due to decreased renal clearance, or higher production of 2Py from NAD^+^ degradation due to PARP activation by means of an experimental design. Conclusions are yet additionally supported by the presence of direct associations of the 24-h urinary excretion of 2Py, *N*^1^-MN, and the sum of 2Py and *N*^1^-MN, but not urinary 2Py/*N*^1^-MN, with niacin nutritional intake (Table S1). Future studies are strongly encouraged to elaborate on serum concentrations of 2Py and *N*^1^-MN along with their urinary excretion. The present study is confined to the urinary excretion of the major NAD^+^ metabolites, comprising the most common and recommended indices of niacin nutritional status according to existing literature and authorities, including the WHO and the EFSA [3,4], respectively. Other indices, including serum or erythrocyte concentrations of niacin and its metabolites [49], are considered inferior as urinary concentrations have shown the most sensitive response to oral test doses of niacin equivalents [3,7]. Given the aforementioned limitations, this study should be conceived as a descriptive report that precludes final conclusions on the applicability of *N*^1^-MN excretion as a biomarker of niacin nutritional status in conditions of impaired kidney function. Finally, although niacin deficiency is considered to be uncommon in the developed world, it might be prevalent in subpopulations, including RTR [12]. Still, it should be emphasized that assessment of niacin nutritional status might not be feasible in the developing world given the costs.

## 5. Conclusions

The 24-h urinary excretion of 2Py is higher in RTR than in kidney donors, and urinary 2Py/*N*^1^-MN clearly increases after kidney donation. Urinary 2Py/*N*^1^-MN is associated with kidney function in both RTR and kidney donors, and kidney function is identified as the strongest determinant of urinary 2Py/*N*^1^-MN in RTR. Therefore, interpretation of both *N*^1^-MN and 2Py excretion, rather than *N*^1^-MN alone, may be recommended for assessment of niacin nutritional status in conditions of impaired kidney function.

## Figures and Tables

**Table 1:** Excretion of 2Py and *N*^1^-MN and kidney function in RTR and kidney donors before and after kidney donation ^1^.

	RTR	Kidney Donors	Population	Kidney Donors		Paired
Variable	*n* = 660	*n* = 275	Difference	*n* = 85		Difference
	Total Cohort	Total Cohort	*p*-Value ^2^	Pre-Donation	Post-Donation	*p*-Value ^3^
Age, years	53.0 ± 12.7	53.3 ± 10.7	0.68	52.2 ± 10.5	52.6 ± 10.4	<0.001
Male, *n* (%)	379 (57)	112 (41)	0.001	43 (51)	43 (51)	-
Body surface area, m^2^	1.9 ± 0.2	1.9 ± 0.2	0.90	2.0 ± 0.2	1.9 ± 0.2	0.01
BMI, kg/m^2^	26.6 ± 4.8	25.9 ± 3.4	0.01	26.0 ± 3.4	25.7 ± 3.2	0.03
Urinary excretion						
2Py, μmol/day	178.1 (130.3–242.8)	155.6 (119.6–217.6)	0.001	152.8 (124.4–215.1)	161.7 (116.6–227.8)	0.31
*N*^1^-MN, μmol/day	22.0 (15.8–31.8)	41.4 (31.6–57.2)	<0.001	40.9 (31.0–58.2)	32.5 (23.4–44.0)	<0.001
2Py/*N*^1^-MN	8.7 ± 3.8	4.0 ± 1.4	<0.001	4.0 ± 1.4	5.2 ± 1.5	<0.001
Sum of 2Py and *N*^1^-MN, μmol/day	198.3 (155.9–269.4)	203.7 (149.4–274.7)	0.98	198.3 (162.3–270.8)	189.7 (141.9–271.6)	0.90
2Py fraction, % ^4^	89.1 (86.4–91.3)	79.0 (75.6–82.1)	<0.001	78.3 (75.5–81.8)	83.5 (80.0–86.0)	<0.001
*N*^1^-MN fraction, % ^4^	10.9 (8.7–13.6)	21.0 (17.9–24.4)	<0.001	21.7 (18.2–24.5)	16.5 (14.0–20.0)	<0.001
Kidney function						
eGFR, mL/min/1.73 m^2^	45.8 ± 18.7	91.0 ± 14.2	<0.001	92.8 ± 13.9	60.1 ± 12.1	<0.001
GFR, mL/min/1.73 m^2^	52.4 ± 17.4	82.3 ± 29.7	<0.001	103.7 ± 16.7	65.3 ± 10.4	<0.001

^1^ Data are presented as mean ± SD, median (IQR) and absolute number (percentage) for normally distributed, skewed and nominal data, respectively. ^2^ *p*-value for difference between RTR and the total cohort of kidney donors was tested by *t* and Mann–Whitney tests for normally and skewed distributed continuous variables, respectively. ^3^ *p*-value for difference between kidney donors before and after kidney donation was tested by paired samples *t* and Wilcoxon signed rank tests for normally and skewed distributed continuous variables, respectively. ^4^ The urinary fraction of 2Py or *N*^1^-MN (percentage) was calculated by dividing 2Py or *N*^1^-MN excretion by the sum of 2Py and *N*^1^-MN excretion, respectively, and multiplying by 100. BMI, body mass index; eGFR, estimated glomerular filtration rate; GFR, glomerular filtration rate; *N*^1^-MN, *N*^1^-methylnicotinamide; RTR, renal transplant recipients; 2Py, *N*^1^-methyl-2-pyridone-5-carboxamide; 2Py/*N*^1^-MN, ratio of 2Py to *N*^1^-MN.

**Table 2:** Associations of urinary 2Py/*N*^1^-MN with kidney function in RTR and kidney donors before and after kidney donation ^1^.

Variable	RTR	Kidney Donors		
		Total Cohort	Pre-Donation	Post-Donation
	*n* = 660	*n* = 275	*n* = 85	*n* = 85
eGFR, mL/min/1.73 m^2^				
Standardized β	−0.40	−0.17	−0.01	−0.11
*p*-value	<0.001	0.03	0.94	0.42
GFR, mL/min/1.73 m^2^				
Standardized β	−0.39	−0.20	−0.02	0.15
*p*-value	<0.001	0.003	0.89	0.27

^1^ Linear regression analyses were performed to investigate associations of urinary 2Py/*N*^1^-MN with kidney function, with adjustment for age and sex. eGFR, estimated glomerular filtration rate; GFR, glomerular filtration rate; *N*^1^-MN, *N*^1^-methylnicotinamide; RTR, renal transplant recipients; 2Py, *N*^1^-methyl-2-pyridone-5-carboxamide; 2Py/*N*^1^-MN, ratio of 2Py to *N*^1^-MN.

**Table 3:** Associations of urinary 2Py/*N*^1^-MN with characteristics in 660 RTR ^1,2^.

Variable	Value	Standardized β	*p*-Value
Urinary 2Py/*N^1^*-MN	8.7 ± 3.8	-	-
2Py excretion, μmol/day	178.1 (130.3–242.8)	-	-
*N*^1^-MN excretion, μmol/day	22.0 (15.8–31.8)	-	-
Age, years	53.0 ± 12.7	0.03	0.09
Male, *n* (%)	379 (57)	−0.004	0.92
Body surface area, m^2^	1.9 ± 0.22	0.11	0.006
BMI, kg/m^2^	26.6 ± 4.8	0.17	<0.001
Creatinine excretion, mmol/day	11.7 ± 3.4	−0.05	0.22
Time since transplantation, years	5.6 (2.0–12.0)	−0.07	0.07
Lifestyle			
Current smoker, *n* (%)	78 (13)	−0.10	0.02
Alcohol consumption, g/day	3.1 (0.0–11.9)	−0.15	<0.001
Nutrition			
Energy intake, kcal/day	2182 ± 642	−0.09	0.03
Niacin equivalents intake, mg/day	35.6 ± 9.2	−0.06	0.14
Vitamin B_6_ intake, mg/day	1.8 ± 0.5	−0.09	0.03
Glucose homeostasis			
Glucose, mmol/L	5.3 (4.8–6.0)	0.15	<0.001
HbA1c, %	5.8 (5.5–6.2)	0.12	0.002
Diabetes, *n* (%)	152 (23)	0.16	<0.001
Lipid homeostasis			
Total cholesterol, mmol/L	5.1 ± 1.1	0.03	0.38
LDL, mmol/L	3.0 ± 0.9	0.07	0.10
HDL, mmol/L	1.3 (1.1–1.7)	−0.20	<0.001
Triglycerides, mmol/L	1.7 (1.2–2.3)	0.17	<0.001
Hemodynamic			
Systolic blood pressure, mmHg	135.8 ± 17.3	0.07	0.08
Diastolic blood pressure, mmHg	82.5 ± 11.0	0.08	0.05
Mean arterial pressure, mmHg	107.0 ± 15.0	0.10	0.02
Inflammation			
Hs-CRP, mg/L	1.6 (0.7–4.6)	0.19	<0.001
Kidney function			
eGFR, mL/min/1.73 m^2^	45.8 ± 18.7	−0.40	<0.001
Proteinuria, *n* (%)	132 (20)	0.08	0.04
Nonimmunosuppressive medication			
Antidiabetic, *n* (%)	96 (15)	0.14	<0.001
Statin, *n* (%)	349 (53)	0.06	0.15
Antihypertensive, *n* (%)	581 (88)	0.09	0.02
Acetylsalicylic acid, *n* (%)	127 (19)	0.09	0.03
Proton pump inhibitor, *n* (%)	326 (49)	0.08	0.04
Immunosuppressive medication			
Prednisolon dose, mg/day	3.0 (2.0–3.0)	0.07	0.07
Proliferation inhibitor, *n* (%)	548 (83)	−0.02	0.71
Tacrolimus, *n* (%)	120 (18)	0.11	0.007
Cyclosporine, *n* (%)	253 (38)	−0.01	0.72

^1^ Data are presented as mean ± SD, median (IQR) and absolute number (percentage) for normally distributed, skewed and nominal data, respectively. ^2^ Linear regression analyses were performed to investigate associations of urinary 2Py/*N*^1^-MN with baseline variables, of which standardized β and *p*-value are presented. BMI, body mass index; eGFR, estimated glomerular filtration rate; HbA1c, hemoglobin A1c; HDL, high-density lipoprotein; hs-CRP, high-sensitivity C-reactive protein; LDL, low-density lipoprotein; *N*^1^-MN, *N*^1^-methylnicotinamide; RTR, renal transplant recipients; 2Py, *N*^1^-methyl-2-pyridone-5-carboxamide; 2Py/*N*^1^-MN, ratio of 2Py to *N*^1^-MN.

**Table 4:** Independent determinants of urinary 2Py/*N*^1^-MN in RTR.

Variable	Univariable		Multivariable ^1^	
	Standardized β	*p*-Value	Standardized β	*p*-Value
Age, years	0.03	0.09	-	-
Male, *n* (%)	−0.004	0.92	-	-
BMI, kg/m^2^	0.17	<0.001	-	-
Time since transplantation, years	−0.07	0.07	-	-
Lifestyle				
Current smoker, *n* (%)	−0.10	0.02	−0.13	0.001
Alcohol consumption, g/day	−0.15	<0.001	−0.12	0.002
Nutrition				
Energy intake, kcal/day	−0.09	0.03	-	-
Niacin equivalents intake, mg/day	−0.06	0.14	-	-
Vitamin B_6_ intake, mg/day	−0.09	0.03	-	-
Glucose homeostasis				
Diabetes, *n* (%)	0.16	<0.001	0.10	0.01
Lipid homeostasis				
LDL, mmol/L	0.07	0.10	-	-
HDL, mmol/L	−0.20	<0.001	−0.12	0.002
Hemodynamic				
Mean arterial pressure, mmHg	0.10	0.02	-	-
Inflammation				
Hs-CRP, mg/L	0.19	<0.001	0.10	0.009
Kidney function				
eGFR, mL/min/1.73 m^2^	−0.40	<0.001	−0.38	<0.001
Proteinuria, *n* (%)	0.08	0.04	-	-
Nonimmunosuppressive medication				
Antihypertensive, *n* (%)	0.09	0.02	-	-
Acetylsalicylic acid, *n* (%)	0.09	0.03	-	-
Proton pump inhibitor, *n* (%)	0.08	0.04	-	-
Immunosuppressive medication				
Prednisolon dose, mg/day	0.07	0.07	-	-
Tacrolimus, *n* (%)	0.11	0.007	-	-
*R* ^2^	0.28		0.26	
Adjusted *R*^2^	0.25		0.25	

^1^ Stepwise multivariable linear regression with backward elimination was performed to identify determinants of urinary 2Py/*N*^1^-MN, of which standardized β and *p*-value are presented. BMI, body mass index; eGFR, estimated glomerular filtration rate; HDL, high-density lipoprotein; hs-CRP, high-sensitivity C-reactive protein; LDL, low-density lipoprotein; *N*^1^-MN, *N*^1^-methylnicotinamide; RTR, renal transplant recipients; 2Py, *N*^1^-methyl-2-pyridone-5-carboxamide; 2Py/*N*^1^-MN, ratio of 2Py to *N*^1^-MN.

## Supplementary Materials

The following is available online at https://www.mdpi.com/2077-0383/9/2/437/s1, Table S1: Associations of 2Py and *N*^1^-MN excretion with niacin equivalents intake in RTR and kidney donors.
