# Supplementary material for: Urinary Excretion of N1-methyl-2-pyridone-5-carboxamide and N1-methylnicotinamide in Renal Transplant Recipients and Donors
Source: J Clin Med. 2020 Feb 6;9(2):437. doi: 10.3390/jcm9020437 (PMC7074074; doi:10.3390/jcm9020437)
Supplement: Supplementary file 1 [file jcm-09-00437-s001.pdf]

Supplementary Materials

Supplementary Table S1: Associations of 2Py and N^1-MN excretion with niacin equivalents intake in RTR and kidney donors [1].

**Variable**	**RTR** *n* **= 660**		**Kidney donors** *n* **= 275**	
Niacin equivalents intake, mg/day	35.6 ± 9.2		37.4 ± 10.8	
	**Standardized β**	***p*-Value**	**Standardized β**	***p*-Value**
2Py excretion, µmol/day	0.22	<0.001	0.24	0.001
N^1-MN excretion, µmol/day	0.25	<0.001	0.29	<0.001
Urinary 2Py/N^1-MN	-0.06	0.14	-0.05	0.52
Sum of 2Py and N^1-MN excretion, µmol/day	0.23	<0.001	0.27	<0.001

[1] Linear regression analyses were performed to investigate associations of urinary excretion of 2Py and N^1-MN with niacin equivalents intake in RTR and healthy kidney donors. N^1-MN, N^1-methylnicotinamide; RTR, renal transplant recipients; 2Py, N^1-methyl-2-pyridone-5-carboxamide; 2Py/N^1-MN, ratio of 2Py to N^1-MN.
